# Self-defense: Deflecting Deflationary and Eliminativist Critiques of the Sense of Ownership

**DOI:** 10.3389/fpsyg.2017.01612

**Published:** 2017-09-21

**Authors:** Shaun Gallagher

**Affiliations:** ^1^Department of Philosophy, University of Memphis, Memphis TN, United States; ^2^Philosophy, Faculty of Law, Arts and Humanities, University of Wollongong, Wollongong NSW, Australia; ^3^Center for Mind, Brain and Cognitive Evolution, Institute for Philosophy II, Ruhr University Bochum Bochum, Germany

**Keywords:** sense of ownership, minimal self, mineness, phenomenology, deflationary account, sense of agency

## Abstract

I defend a phenomenological account of the sense of ownership as part of a minimal sense of self from those critics who propose either a deflationary or eliminativist critique. Specifically, I block the deflationary critique by showing that in fact the phenomenological account is itself a deflationary account insofar as it takes the sense of ownership to be implicit or intrinsic to experience and bodily action. I address the eliminativist view by considering empirical evidence that supports the concept of pre-reflective self-awareness, which underpins the sense of ownership. Finally, I respond to claims that phenomenology does not offer a positive account of the sense of ownership by showing the role it plays in an enactivist (action-oriented) view of embodied cognition.

## Introduction

A growing army of theorists have struck out in attack mode against the notion of a pre-reflective, minimal sense of self or sense of ownership (e.g., Dainton, 2008, 2016; Bermúdez, 2011, in press; Prinz, 2012; Di Francesco et al., 2016; Garfield, 2016). To defend against such attacks I’ll follow a divide and conquer strategy. Specifically, in this paper I take on the deflationary and eliminativist arguments advanced by Bermúdez, Dainton, and Di Francesco and colleagues.^1^ My tactic will be to use a form of philosophical Jujitsu. That is, I’ll use the power of the critics’ own arguments against them by showing (1) that the pre-reflective sense of ownership is, on the phenomenological accounts that are criticized, intrinsic to experience, rather than some additional quality, and this is just what the deflationary account requires; (2) that the concept of a pre-reflective sense of ownership is consistent with the empirical evidence that the critics themselves cite; and (3) that a more positive account of the sense of ownership is to be found in the phenomenologically inspired enactivist (action-oriented) view of experience as always embodied and most often agentive.

The notion of the sense of ownership (SO) is a complex one. First, the phrase itself may be misleading. The term ‘ownership’ typically applies to the ownership of things, objects, or property and tends to signify a legal claim about such property. Clearly, one’s body is not piece of property, except perhaps in a metaphorical sense. We are not in this kind of relationship of ownership to our own body. One can agree with Bermúdez on this point; he suggests that “ownership is a rather tenuous and metaphorical concept in this context. We do not own hands …… in the way that we own personal property” (in press). Although there are contexts in which the concept of legal ownership of one’s body may be appropriate,^2^ in the context that we are considering here, which involves a phenomenological conception, this would be a misunderstanding. In this context, however, we find the phrase ‘sense of ownership’ in use at least from the mid-1990s, for example in Martin (1995), who defines it as “the phenomenological quality that [a] body part appears to be part of one’s body” (Martin, 1995, p. 269; see Martin, 1992). Moreover, this concept, if not this specific phrase, had been discussed by earlier philosophers, such as Husserl, and in the phenomenological psychiatry tradition following Jaspers. Rather than the term ‘ownership,’ however, phenomenologists tend to use the term *mineness* or the *experience of mineness* (see, e.g., Hopkins, 1993).

Importantly, SO or the sense of mineness, applies not only to one’s body or one’s body parts; it also applies to movement, action, and even to experience itself. I may have a sense that this is *my* action, or *my* thinking, or, most basically, *my* experience (Gallagher, 2000). Guillot’s (2017) offers some clarification on this by distinguishing between three phenomena.

• **For-me-ness** – the awareness of the *experience* as I live through it
• **Me-ness** – a pre-reflective self-awareness that *I* am the one living through the experience
• **Mineness** – the sense that this is my experience (ownership), i.e., an awareness of the experience as my own.

Guillot argues that in non-pathological/normal experience we have all three.

It is something about the experience, something intrinsic to it, that supports judgments [about the experience]. This I take to be at least a prima facie reason to think that we typically have experiential access to the experience, to ourselves, and to the fact that the experience is ours; or, in my terminology, that the phenomenal character of a normal experience includes for-me-ness, me-ness, and mineness (Guillot, 2017, p. 47).

I think it is quite possible to accept these distinctions as conceptual distinctions, without thinking that we actually experience such distinctions, or that these differences are experienced as such. On the phenomenological view, the experience is precisely the experience of mineness (ownership), which is an intrinsically relational experience, i.e., involving the relation between me (as experiencer) and the experience itself, rather than an experience of a relation. For-me-ness and me-ness are, accordingly, abstractions from mineness that I can make in reflective judgment.

Guillot also argues (following Billon, 2011) that in some pathological cases mineness (or the sense of ownership) goes missing. Without entering the various debates about depersonalization and schizophrenic delusions of control and thought insertion, however, there are two important points to take from the idea that SO is missing in such exceptional or pathological circumstances. First, it implies that SO is clearly not a necessary or essential aspect of all experience, but also, second, it implies that SO is present in everyday normal experience.

The phenomenological view is that SO/mineness is experienced in the pre-reflective (or non-reflective) self-awareness that is intrinsic in everyday (non-pathological, non- exceptional) conscious experience. It is experienced as this pre-reflective self-awareness and is nothing over and above this pre-reflective self-awareness (Gallagher and Zahavi, 2012, 2014). Nor does it require an extra or transitive act of self-awareness which takes experience as an object. Accordingly, it is also important to distinguish this first-order, pre-reflective SO from a retrospective (reflective) judgment about ownership (Vosgerau and Newen, 2007).

## The Deflationary Account

Martin’s (1995) use of the phrase ‘sense of ownership’ was meant to describe an experience of one’s spatial boundaries. According to Martin, “when one feels a sensation, one thereby feels as if something is occurring within one’s body” (p. 267). This is not a matter of explicit judgment, as if I were experiencing a free-floating sensation concerning which I needed to judge its spatial location as falling within my body boundaries. Rather, as Martin argues, the experience of location is an intrinsic feature of the sensation itself. This experience just is the SO for one’s body as a whole, so that I have SO for particular body parts only as being parts of that whole body (Martin, 1995, pp. 277–278). In this regard, SO is not a quality in addition to other qualities of experience, but “already inherent within them” (p. 278). This is consistent with the phenomenological view: SO is an intrinsic aspect of proprioceptive and kinaesthetic experiences of bodily movement, and other bodily sensations.^3^

Bermúdez (2011, in press), however, in a critical discussion of SO, in contrast to Martin, rejects the idea that SO is a “special phenomenological relation” (Martin, 1995, p. 267), although he accepts the importance of “boundedness.” He denies that there is a positive first-order (non-observational) phenomenology of ownership or feeling of ‘mineness.’ In contrast to what he calls an “inflationary” conception, which he attributes to phenomenologists like Merleau-Ponty (he also cites Gallagher, 2005; de Vignemont, 2007, 2013), he offers a deflationary account. “On a deflationary conception of ownership the sense of ownership consists, first, in certain facts about the phenomenology of bodily sensations and, second, in certain fairly obvious judgments about the body (which we can term judgments of ownership)” (Bermúdez, 2011, p. 162). His deflationary view is that an explicit experience of ownership only comes up when we turn our reflective attention to our bodily experience and attribute that experience to ourselves. This, I think, is the only way to make sense of his claim that “[w]hen we experience our bodies we experience them as our own … there is a phenomenology of ownership” (Bermúdez, 2015, p. 38). I take this to mean that there is a second-order phenomenology of ownership derived from the judgment of ownership.^4^ A second-order experience of ownership results as a product of this judgment, but it is not something that is there to begin with. “There are facts about the phenomenology of bodily awareness (about position sense, movement sense, and interoception) and there are judgments of ownership, but there is no additional feeling of ownership” (Bermúdez, 2011, p. 166).

Bermúdez thus comes close to the eliminativist view, since he rejects the idea that there is “a specific feeling of ownership – a qualitative ‘feel’ that one has in all and only those body parts that one experiences as one’s own” (in press). According to Bermúdez (2011) SO is a philosophical fiction. Although one does experience a sense of body boundedness and connectedness, one does not experience, in addition, SO as a separate and independent feeling.

As we’ve seen, however, for the phenomenologists, to say that SO is an intrinsic aspect of proprioceptive and kinaesthetic experiences is to agree that it is not an additional or independent feeling, but rather, a sense “already inherent within” the phenomenology of bodily sensations. On the phenomenological view, and in contrast to Bermúdez, this intrinsic aspect is pre-reflective in the sense that one has this intrinsic experience of ownership without having to make a reflective judgment about ownership. This can be read in the deflationary way, so that the phenomenologists can agree that there is no additional feeling of ownership, or “perfectly determinate ‘quale’ associated with the feeling of myness” (Bermúdez, 2011, p. 165), independent of the proprioceptive and kinaesthetic sensations. In contrast to the eliminativist view, however, there is still an experiential SO. In fact, this implicit self-experience is precisely what makes first-person bodily (proprioceptive, kinaesthetic) awareness itself (i.e., prior to any judgment) a form of self-consciousness. It’s what puts the ‘proprio’ in proprioception (Gallagher and Trigg, 2016).

Bermúdez doesn’t want to deny, however, that we can have a proprioceptive and kinaesthetic awareness of bodily (and limb) posture and movement. This is clear in his (2015) discussion of an example from Anscombe (1962). Anscombe considers the meaning of the expression ‘sensation of X’ in the example of the sensation of going down in a lift. Does the phrase ‘the sensation of going down in a lift’ signify (1) the subjective feeling I have of an upward feeling in my stomach (the internal description or content of the sensation), or (2) the objective event of going down in a lift (the external accompaniment). Even if my focus of attention is on the objective event, that focus carries with it a non-reflective (non-observational) awareness of my phenomenal experience (the sensation content) which includes, implicitly, the sense that this is happening to *me*. This is a non-reflective self-awareness that, roughly, it is *my* stomach that is moving upward, or perhaps something more indeterminate, but nonetheless, an experience in or of *my* body. Bermúdez endorses the idea that such experiences give us a sense of boundedness and connectedness “from the inside.” Throughout such proprioceptive experiences, however, there is a more or less integrated pattern of experience in which body awareness includes an intrinsic experience that it is *my* bodily experience. That’s the *proprio* in proprioception.

It’s not clear what this kind of awareness could be, other than a pre-reflective awareness that is built into (not something separate and distinct from) the structure of precisely the experience I have of my body or of a sensation that is located in my body. This idea is consistent with both the phenomenological view, and the deflationary account offered by Martin. This is also what Dokic (2003) claims in an account that Bermúdez identifies as deflationary. “Bodily experience gives us a *sense of ownership….* The very idea of *feeling* a pain in a limb which does not seem to be ours is difficult to frame, perhaps unintelligible” (Dokic, 2003, p. 325).

## The Sense of Self in the Phenomenal Background

Dainton (2008), in his discussion of what he terms the “isolation thesis,” i.e., the idea that there could be just one isolated bodily sensation, e.g., of pain, takes issue with the phenomenological concept of pre-reflective self-awareness. Typically, in contrast to the isolation thesis, when I experience some sensation I experience it “against the backdrop of various other forms of consciousness: a range of bodily experience, tactile sensations, visual and auditory experience, intentional or willed bodily movements, conscious thinking … [etc.]” (2008, pp. 239–240). This backdrop of experience, to which we are not attending when we attend to the pain, he calls the ‘phenomenal background.’ This background consists of two regions – a worldly region where I experience, e.g., in exteroception, the sights and sounds around me, and an “inner” region, an elusive set of bodily experiences, thoughts, memories, and so on. He suggests that this inner aspect of the phenomenal background contributes to (and perhaps constitutes) “the feeling of what it is typically like to be *me* (or *you*)’ (p. 240). This inner background may be relatively stable, as Dainton suggests, but it does not consist of a particular kind of sensation or feeling. Specifically, he argues, it does not consist of a pre-reflective self-awareness or sense of mineness or ownership.

I can see no reason to take this stability as indicative of a single special type of experience, something over and above the changing stream of thought, perception, volition, emotion, memory, bodily sensation, and so on (p. 240).

He argues that if we subtract all of these various experiences, there would be nothing of experience left; therefore, there is nothing over and above just these experiences – no extra or additional experience that we would identify as the experience of mineness. Rather, he suggests, the “ambient ‘sense of self”’ is something like the product of all of these experiences. This phenomenal background is always something of which we are co-conscious, but always something precisely in the background, and of which we are not *explicitly* aware. It’s this ubiquitous presence of the phenomenal background – this ambient sense of self – that makes it impossible to imagine an “ownerless” isolated pain sensation.

Dainton takes this argument to undermine the phenomenological claim that there is a particular form of self-consciousness, of the minimal or ‘non-reflexive’ variety, that always accompanies experience. Zahavi, whom Dainton quotes, defends this sort of phenomenological claim.

One commonality [shared by all experiences] is the quality of *mineness*, the fact that experiences are characterized by first-person givenness. That is, the experience is given [i.e., experienced] (at least tacitly) as *my* experience, as an experience *I* am undergoing or living through…. Phenomenal consciousness must be interpreted precisely as entailing a minimal or thin form of self-awareness. On this account, any experience that lacks self-awareness is non-conscious (Zahavi, 2005, p. 16; cited in Dainton, 2008, p. 242).

Dainton pushes back against this claim. “There is certainly no obvious need to posit a quality of *mineness* to explain how it is that we are always aware of our own experiences” (2008, p. 242). Rather, Dainton holds that experiences are intrinsically conscious and as such they “automatically contribute to the overall character of their subject’s consciousness,” without any “further assistance” by an additional quality of mineness. The phenomenologists may be right that I, as experiencing subject, am not usually in doubt about who the subject of my experience is, and again Dainton cites Zahavi: “Whether a certain experience is experienced as mine or not, however, depends not on something apart from the experience, but precisely on the givenness of the experience” (2005, p. 124). But then Dainton goes on to ask: “do we need *mineness* to explain whether an experience is experienced as mine?” (2008, p. 242). It’s not that Dainton doubts that one’s experience is something that one is aware of living through – it’s just that he doubts that we need the additional experience of mineness to make it so. Rather, the sense of self is given by the phenomenal background.^5^

Two things follow from Dainton’s analysis.^6^ “First, we can account for the phenomenology of *mineness* without positing any primitive ‘ownership’ quality” (p. 243). And second, a reductionist view of our sense of self is possible – that is, “our sense of self is not the product of a single simple form of experience, but rather the joint product of several different sorts of (quite ordinary) experiences” (p. 243).

If we accept Dainton’s argument, then there does exist a sense of mineness or SO, but it is not a special or additional quality, or a primitive pre-reflective self-awareness added to the phenomenal background. Does this actually constitute an argument against the phenomenological concept of sense of mineness or ownership? It’s difficult to see how it would count against the phenomenological conception since the phenomenologists, including Zahavi, describe the sense of mineness as an *intrinsic* aspect of experience, not as something extra that is added, or an additional quality that one experiences in addition to experiencing pain, or bodily sensations, or thinking, etc. To repeat Dainton’s quotation from Zahavi: “Whether a certain experience is experienced as mine or not, however, depends not on something apart from the experience, but precisely on the givenness of the experience” (2005, p. 124). As Zahavi most recently put it:

the what-it-is-likeness of phenomenal states is properly speaking a what-it-is-like-for-me-ness. On this view, experiential processes are *intrinsically* conscious and hence self-revealing. They are characterized by an *inherent* reflexive (not reflective) or pre-reflective self-consciousness in the weak sense that they are like something for the subject, i.e., in virtue of their mere existence, they are phenomenally manifest to the subject of those experiences (Zahavi, in press; emphasis altered).

Likewise, Gallagher and Zahavi (2014) emphasize the intrinsic or inherent nature of pre-reflective self-awareness.

Experience happens for the experiencing subject in an immediate way and as part of this immediacy, it is implicitly marked as *my* experience…. [P]re-reflective self-consciousness is pre-reflective in the sense that (1) it is an awareness we have before we do any reflecting on our experience; (2) it is an implicit and first-order awareness…. The mineness in question is not a quality like being scarlet, sour or soft. It doesn’t refer to a specific experiential content, to a specific what; nor does it refer to the diachronic or synchronic sum of such content, or to some other relation that might obtain between the contents in question. Rather, it refers to the distinct givenness or the how it feels of experience…. That pre-reflective self-awareness is implicit, then, means that I am not confronted with a thematic or explicit awareness of the experience as belonging to myself. Rather we are dealing with a non-observational self-acquaintance.

‘Intrinsic’ means that it is built into the structure of such experiences,^7^ not something added on. That it is an intrinsic aspect of the phenomenal background is not something that phenomenologists would disagree with. It is not clear, however, that one should regard it as the “product” of the experiences that make up the phenomenal background, as Dainton suggests, since that way of putting it actually implies that it is something in addition to those experiences. As I understand it, however, that is not Dainton’s intended claim, and in this respect (*pace* his critique) he is in agreement with the phenomenological conception of the sense of ownership.

Accordingly, for both Dainton and Bermúdez, the force of their arguments against the phenomenological conception of the SO as a form of pre-reflective self-awareness intrinsic to experience can be turned around and redirected to show that in fact the phenomenological conception is precisely the conception that they need in order to make sense out of their own views, at lease if we take them to be defending deflationary views.

## The Eliminativist Account

I suggest that a similar philosophical Jujitsu can also be used against the more sustained critique offered by Di Francesco et al. (2016). They argue (citing Schear, 2009) that by using a contrastive strategy we can find states of consciousness that *prima facie* lack any kind of self-consciousness. Specifically, they cite experiences involved in meditative trance and high-level athletic performance.

In these kinds of mental states, we are completely immersed in a certain task and forgetful, so to speak, of ourselves. We are one and the same thing with a certain thing or task. However, this strategy is not available to Gallagher and Zahavi, since it implies that there are conscious but non-self-conscious states, whereas, according to these authors and their followers, mineness is a *necessary* ingredient of consciousness (2016, p. 79).

First, let me block the thrust of their point about meditative trance. Perhaps it’s an open question, but it is not at all clear how one can report on that state of consciousness if in fact at the time of that experience one is not, at least, pre-reflectively aware that one is in that state of consciousness. And if it is a retrospective report, it implies that there was some minimal self-awareness present, and that the subject registered the experience as his or her own; otherwise, it’s not clear how or why he or she would be reporting it. Indeed, according to MacKenzie (2008), reflexivist or self-illumination (*svaprakāśa*) theories in classical Indian philosophy defend the idea that if a state is conscious, it is simultaneously consciousness of both the object of consciousness and the conscious state itself. In this respect, it is like Hume’s famous claim not to be able to find a self among his experiences. But the fact that he looks among *his own* experiences, rather than anyone else’s (and it could not be otherwise), and reports it as such (“whenever I enter into what I call *myself*”), suggests that there is some kind of implicit self-awareness that these are *his* experiences. In any case, in regard to the claim about meditative trance, it’s not clear what the evidence is.

Second, the evidence against Di Francesco and colleagues, claim may be clearer with respect to high-level athletic performance, since there are studies that show that in such flow-like performance there still is some kind of pre-reflective self-awareness involved [e.g., Christensen et al., 2016; likewise, in dance (Legrand, 2007; Montero, 2012, 2016) and in flow-like states during musical performance (Høffding, 2015; Salice et al., 2017)]. Furthermore, as we noted with respect to pathological experience, if Di Francesco and colleagues were right about meditative trance and exceptional performance, the implication is that these experiences, at the very least, would be exceptions to the rule that everyday (non-exceptional) consciousness actually does involve an implicit pre-reflective self-awareness. The exceptions contrast to the more general fact of the matter – otherwise they would not be so exceptional. Yet, this is clearly not what Di Francesco and colleagues intend. Indeed, they suggest there is “an extreme difficulty” in finding mineness in experience, in contrast to the phenomenological claim that it would be difficult (if not impossible) to find instances in which experience was without a sense of mineness. Since they do indicate that this is an empirical issue,^8^ the best way to address this conflict of intuitions would be to cite empirical evidence. Di Francesco and colleagues, however, question some of that evidence.

For example, they reject the idea that studies of neonate imitation can offer any evidence that young infants have a pre-reflective sense of mineness. They consider any such appeal to involve an adultist interpretation of infant experience.^9^ Despite being fans of both contrastive strategies and operationalizing phenomena, however, they ignore the precise operational definition of neonate imitation that Meltzoff and Moore (1977) adopt, namely differential imitation. Instead, they accept the account given by Jones (2009), which ignores the operational definition and treats tongue protrusion as simple arousal. The point for Di Francesco and colleagues is that if neonate imitation were just arousal, then there would be no proprioceptive awareness necessary – and so, no SO. The operational definition of neonate imitation adopted by Meltzoff and Moore, however, is not the production of one gesture more often than an unrelated one (as Keven and Akins, 2016, suggest). Rather, they, and the majority of neonate imitation studies, operationalize imitation as “the greater frequency of a gesture in response to the same gesture than in response to other gestures” (Vincini et al., in press, 2017). The operational definition entails reference to a plurality of gestures exhibiting a comparative increase. As Meltzoff and Moore were well aware, this is a crucial point since if only one gesture is matched, then arousal would be the most plausible explanation; the operational definition of differential imitation was meant to exclude the arousal explanation. This makes it an empirical question: is there evidence for differential imitation? A recent study by Coulon et al. (2013), for example, provides evidence for differential imitation. A more recent study, Oostenbroek et al. (2016), fails to provide evidence for differential imitation, but that may be because they employed highly conservative criteria. If this question remains unsettled in the literature, then the jury is still out, and Di Francesco and colleagues cannot simply help themselves to their preferred account.

In regard to the question of pre-reflective proprioceptive self-awareness, the relevant aspect of what Meltzoff and Moore showed was that that the infant’s imitation improved with practice, implying that the infant was able to discriminate between its own facial gesture and the gesture it saw on the other’s face. This was regarded as evidence for a basic, proprioceptive-based distinction between self and non-self in newborns (see, e.g., Bermuúdez, 1996; Gallagher, 1996).^10^ But if Di Francesco and colleagues are not happy with the evidence from neonatal imitation, that is not the only place one can find evidence for this basic distinction. One can find it in turn-taking in proto-conversation, and differential kinematic responses to self versus non-self (Reddy, 2008). Even the fetus can discriminate the difference between being moved and moving itself (Glass, 2005). The differentiation is also built into touch, so that the sensory-motor system of the infant can register the difference between someone else’s hand touching its face (eliciting the rooting reflex) and it’s own hand touching its face (no rooting reflex) (Rochat and Hespos, 1997). Even prior to birth the physiological requisites for proprioception are in place (Humphrey, 1964; Van der Meer et al., 1995).

This kind of evidence is, of course, open to interpretation. On the one hand, Di Francesco and colleagues might argue that all of this differentiation between self and non-self is really non-conscious. Whether or not that is the case for the late-term fetus, however, it’s not clear why conscious discrimination in the infant would need to wait until 4–5 months, as Di Francesco and colleagues suggest. Indeed, Di Francesco and colleagues they cite Bermúdez’s view on this: “Somatic proprioception and the structure of exteroceptive perceptual experience can be a source of non-conceptual first-person contents from the very beginning of life” (Bermuúdez, 1998, p. 163; cited by Di Francesco et al., 2016, p. 72). If the structure of perception contains propriospecific information, for example, as the boundary of the visual field that originates in the embodied perspective of the agent, that just is the basis for the inbuilt structure of pre-reflective self-awareness. As Di Francesco and colleagues note, on this ecological view, “affordances, visual kinestesis and bodily invariants all carry self-specifying information” which is “precociously available to the child” (p. 83).

Although Di Francesco and colleagues don’t reject these ecological claims, they do reject the idea that such experience could be taken as evidence for pre-reflective self-awareness. Why? I think we get to the real punch of their view here. They reject ecological experience as a form of pre-reflective self-awareness in younger infants because those younger infants do not yet have a more advanced objective experience of their body as a whole. First, they claim that ecological self-awareness is awareness of “single parts of the body, not of the body taken as a whole” (p. 84). Yet ecological self-awareness may very well be awareness that I am moving through the environment, for example, by walking or running, or that I am sitting or standing still. It’s not at all clear that such awareness is focused on one or several body parts rather than the entire body.

Second, they claim that “when a baby, say, 6 or 8 months old perceives, say, her hand, she perceives it *as an object among others*, not as *part* of her body” (p. 84); to perceive it as part of her body she would have to be able to represent her body as a whole, which is not yet possible, according to Di Francesco and colleagues. But isn’t this move already blocked by our previous considerations? Although it may be clear that the child can indeed take an objective view of her hand, and in this regard does not have immunity to error with regard to identifying her hand as *her* hand, it should also be clear from the evidence cited above, and from the very nature of proprioception and kinaesthesis, the child often and usually does have an agentive, first-person experience of her hand as her own – as the one she is actively moving, for example (Gallagher, 2015). Phenomenologists refer to this as the body-as-subject (e.g., Legrand, 2007) or the body-as-agent (the *Leib*), which is associated with body-schematic processes, in contrast to body image, the body-as-object, or the objective perception of one’s body (Gallagher, 2005). On any account of SO that involves agentive body-schematic processes rather than body image, the role of the body-as-object can only be secondary or accessory (see, e.g., de Vignemont, 2007, in press). I’ll come back to this point in the final section.

Di Francesco and colleagues claim, then, is that before an agent can have a sense of her own body-as-subject – before she is able to sense that her whole body is moving as she crawls around or starts to walk – she must have developed a body image for her whole body. She apparently just doesn’t have a sense that this hand is her hand until a point in development when she has a developed body image for her whole body. Thus, “we can say that the newborn, like the infant at 6 months or 1 year of age, produces a rich subjectivity, but being immersed in it, cannot objectify it” (p. 85). Likewise for pain: the infant experiences pain, but does not objectify herself as being in pain. For the infant to be able to experience pain as *her own* pain, or her movement as *her own* movement, or her body (or body part) as *her own* body, she requires a developed, objective bodily self-consciousness which comes, according to Di Francesco and colleagues at around 18 months with mirror self-recognition when the child is able to form a body image of herself as a entire object and associate this with herself as a subject – “the active source of the representation” of herself (p. 85).

Di Francesco and colleagues claim here is largely definitional. That is, they define self-consciousness precisely as an explicit *objective* view of “the whole body of the organism [objectively] *experienced as one’s own body*” (p. 85), and nothing less. On the one hand, of course, if we accept this as the exclusive definition of self-consciousness, then nothing like a pre-reflective self-awareness exists, full stop. On the other hand, to say that there may be a developed perspective where one is able to make an objective judgment about bodily ownership, is not to show that self-specifying proprioceptive/kinaesthetic information does not provide a pre-reflective sense of ownership for one’s experience, one’s body, and/or one’s action. A more developed higher-order (conceptual and objective) form of self-consciousness remains consistent with the existence of a pre-reflective sense of ownership. Indeed, on some accounts, one requires just such a proprioceptive sense of one’s own body to be able to recognize it in the mirror. To opt for a purely higher-order conception of self-consciousness is just to endorse a definition that, as Rochat and Zahavi (2011, p. 206) put it, has “dramatic implications … for our ascription of an experiential life to infants,” and opens a larger set of questions, for example, about social cognition, than can be explored here. In this respect, if opting for a more objective conception of self-consciousness was meant to be a knockout blow to the concept of a pre-reflective SO, Di Francesco and colleagues are unable to land their strongest punch.

## Action-Oriented Ownership

Di Francesco and colleagues suggest that phenomenologists fail to offer a positive account of pre-reflective SO, and that it is “characterized only negatively” and without reference to the capacities or processes of self-consciousness (2016, pp. 86–87). One might also think that a deflationary account, that takes SO to be nothing over and above experience itself, will not give us a positive account of this phenomenon. In this final section I want to argue that phenomenologically inspired enactivist approaches to experience and action, specifically involving the agentive body (or the body-in-action), do provide a positive account of SO in terms of the capacities and processes of self-consciousness.

One concern directly related to action is about reliability and precision. In this regard, it is important to note that *bodily* awareness, i.e., awareness not just of the body (body awareness), but awareness of the world that includes pre-reflective self-awareness of my active moving body, following the ecological view, is multimodal (see, e.g., Gallagher, 2005; Tsakiris, 2016). There is good reason for this. Proprioceptive awareness of one’s body is attenuated and not overly precise. The attenuation would be a “flaw” (de Vignemont, 2014, p. 998), however, only if one assumed that proprioception was supposed to deliver precise awareness of the objective body. Proprioception, or any other bodily sense, however, never functions just by itself (Dainton is right about this), and reliability should be measured in terms of the whole system and its integrated functioning. Moreover, we should consider questions about reliability and precision in pragmatic (action-oriented) terms rather than in epistemic terms. In this case, proprioception, functioning along with other modalities (touch, vision, interoception, etc.), provides a pragmatic bodily awareness related primarily to the subject’s action possibilities. The fact that, as Vignemont notes, it does not give me a precise sense of my bodily posture, or shape, or boundaries as I am lying in bed or am not moving, in contrast to when I am moving, is not a problem since accuracy in such circumstances is not that important. Even in regard to action, I do not always need precision information about body boundary or limb location, and, as I’ve suggested elsewhere (Gallagher, in press), we get enough precision when we need it *via* the mix of senses, and pragmatic estimates are good enough in most cases. Indeed, if we take precision to mean objective position sense, this is not something we need for most of our actions.

The enactivist point in this is that bodily experience, in the form of proprioception, kinaesthesia, interoception, etc. is action-oriented. In the same way that perception is enactive, that is, oriented to the possibilities or affordances for action and for responding to others, the proprioceptive-kinaesthetic, pre-reflective SO contributes to how the body attunes to what it can do. Proprioception, as position sense, i.e., as a positive sense of where my limbs are (and the *mineness* of those limbs being an implicit but still positive experience), is not simply the registration of where my limbs are for the sake of knowing where my limbs are, as if it were solving an epistemological or theoretical problem. Rather, it addresses a pragmatic problem: if I want to pick up a hammer, I don’t first of all have to go looking for my hands – they are already ready to go.

In typical, everyday experience, SO readily integrates with a sense of agency (SA) and in most cases SA and SO are experientially indistinct, a fact that is consistent with the deflationist account of SO and with an embodied enactivist conception of SA (see Tsakiris et al., 2007; Gallagher, 2012, 2013; Buhrmann and Di Paolo, 2015). Langland-Hassan (2008), for example, suggests that the phenomenology of agency is “one that is embedded in all first order sensory and proprioceptive phenomenology as diachronic, action-sensitive patterns of information; it does not stand apart from them as an inscrutable emotion” (p. 392). Again, this is fully consistent with the phenomenological view.

Not only does SO play a role in everyday pragmatic actions, it also serves communicative actions and social interactions. This is not a topic that I will develop in detail here (see Gallagher, 2005, 2017), but I want to at least give some indication of what this role is. de Vignemont (2007) suggests that you experience SO for your hand when another person touches it, in a way that you do not feel SO for the touching hand. In this regard, Merleau-Ponty’s well-known example of one hand touching another, as an example of what he calls ‘reversiblility,’ can help to show that there is a potential for action (something action-oriented) in the experience of my hand being touched, and not just an experience of bodily location.

Merleau-Ponty’s example is first of all about my own two hands. If I use my right hand to touch my left hand, there is the immediate possibility of a reversibility – that my right hand touching can immediately become the touched; and my left hand touched can immediately become the touching. If the touching-touched is in some objective sense simultaneous, in terms of our single-minded attention it is not; it involves a dynamic sequential reversibility, not unlike the reversing of the Necker cube in vision, but one that can be easily done at will (Merleau-Ponty, 1968, p. 141). My attention can go back and forth between touching and being touched, attempting to capture a structure that is pre-reflectively already established at the sensory level. Each hand, whether touching or being touched holds a relation to action, something actualized in the case of touching, but only potential in the case of being touched. Even as my one hand is touched, it holds a certain power for touching which could reverse the action^11^.

This is the case whether it is my own hand touching my other hand, or, as in Vignemont’s example, someone else’s hand touching mine. Indeed, Merleau-Ponty suggests that something similar manifests itself in social interaction: “when touching the hand of another, would I not touch in it the same power to espouse the things that I have touched in my own?... [T]he handshake too is reversible; I can feel myself touched as well and at the same time as touching” (1968, pp. 141–142). I suggest that these very basic, embodied contingencies, which Merleau-Ponty associates with the phenomenon of ‘intercorporeity’ (2012, pp. 190–191; Gallagher, 2016), play a positive role in communicative turn taking as well (Reddy, 2008). In the sensation of being touched there is, along with a sense of location and boundary, and the implicit SO that comes along with this, a sense of agency to the extent that I have control over the reversibility – in effect, to the extent that I can immediately turn the being touched into an act of touching. This sense of agency, tied to my potentiality for action and interaction, just to the extent that it is *my* hand that is involved, is integrated with SO.

Given that the whole body can move and can touch or be touched, this applies not just to hands. Likewise, this is not just about proprioception. A pain in my leg can define what I can and cannot do, and can diminish my sense of agency or potential for action in the world. Interoceptive aspects of hunger or fatigue may do the same. Proprioception, however, is important not only for registering the location or position sense of my body. Proprioception also plays a role in motor control, and without proprioception we lose control over our body, and this can diminish SA as well as SO^12^.

There is one final point to be made in regard to a positive action-related characterization of SO. The principle experimental paradigm for studying a bodily sense of ownership is the Rubber Hand Illusion (RHI) (Botvinick and Cohen, 1998). It turns out, however, that SO’s agentive function does not play a clear role in the experience of the RHI. Although we can be tricked into experiencing the rubber hand as our own, if the illusion is maintained, the rubber hand seemingly plays no role in action preparation; ownership seemingly serves no agentive function. Vignemont, however, proposes an alternative agentive role for SO that does persist through the RHI, namely, a self-defense role that retains the connection between SO and body schematic processes, and does not reduce SO to a pure body image phenomenon. Rather than focus on the goal-directed instrumental movements (pointing and grasping) that are tested (albeit infrequently) in the RHI, she suggests that there is a “different range of movements … worth exploring, namely defensive movements” (in press).

[I]t has been repeatedly shown that participants react [defensively] when the rubber hand is threatened, but only when they report it as their own after synchronous stroking, and the strength of their reaction is correlated with their ownership rating in questionnaires (Ehrsson et al., 2007).

Vignemont thus suggests that SO has “a specific agentive mark in the context of self-protection.” One might think that this function is even more evolutionarily basic than SO’s function in instrumental and communicative actions. It’s clear, however, that the sense of ownership, described as a pre-reflective bodily self-awareness, can play important and positive agentive roles in action, communication, and self-defense – and this idea is not something that we should give up without a fight.

## Author Contributions

The author confirms being the sole contributor of this work and approved it for publication.

## Conflict of Interest Statement

The author declares that the research was conducted in the absence of any commercial or financial relationships that could be construed as a potential conflict of interest.

## Footnotes

1 I’ll focus on the eliminativist views expressed in Bermúdez and defended by Di Francesco and colleagues. Prinz (2012) also offers what can be considered an eliminativist proposal – that there just is no experiential sense of self. Likewise, Garfield (2016, p. 73) claims, there is “nothing that it is like to have qualitative experience.”

2 Petchesky (1995) for example, traces the use of the concept of body ownership through a number of legal and historical contexts, and discusses a “shift in the early-modern European origins of ideas about owning one’s body” which has less to do with property rights in an economic sense and more to do with claims about protecting “one’s sexuality and personal security from arbitrary invasion” (p. 390). Also see, e.g., Pateman (1988) for use of this term in feminist discussions.

3 de Vignemont (in press), suggests some qualifications to Martin’s analysis by considering cases in which there is no SO for a bodily limb although sensations may register on that limb. In these cases, e.g., somatoparaphrenia and the case of IW, it is important to note that proprioception/kinaesthesia is missing (Gallagher and Cole, 1995; Vallar and Ronchi, 2009). Accordingly, for a more precise characterization of SO in terms of body boundaries, one should define such boundaries as proprioceptive.

4 See Alsmith (2015). Also, de Vignemont (in press) attempts to clarify this by distinguishing between a ‘feeling of myness’ understood as a first-order experience, which she associates with the inflationary account; and a sense of ownership as a second-order phenomenology, which Bermúdez associates with the deflationary account. I am not distinguishing between a sense of mineness and a sense of ownership – I treat both phrases as signifying a first-order aspects of experience distinguishable from the judgment of ownership.

5 I note that for Dainton, the sense of self is being equated with mineness or the sense of ownership. As one reviewer pointed out, these are at least conceptually distinct, in that a sense of self could be defined as a very basic form of self-consciousness (e.g., sensitivity to self-specific information) without a sense of ownership. What Dainton denies is that the sense of self or mineness is something separate from the phenomenal background.

6 I leave aside the specifics of his argument about the isolation thesis – he does think that it is difficult to rule out the idea that we might be able to experience an “isolated and *phenomenologically ownerless*” sensation, i.e., that the phenomenal background might in fact go missing.

7 That it is built into the structure of experience is explained by Husserl, and the phenomenologists who follow him, in terms of the temporal structure of experience – the retentional-protentional structure that characterizes all of our typical experiences. It would take us too far a field to sketch this analysis, but see Gallagher (1998) for more detail.

8 Di Francesco and colleagues also indicate that no one is making a transcendental claim in this regard. They go on, however, to suggest that the claim made by phenomenologists may be *a priori* (2016, p. 79) or “heavily dependent on *a priori* assumptions” which they associate with transcendental phenomenology (p. 78). To suggest that the phenomenologists’ claim is *a priori*, however, is to misconstrue the nature of phenomenology. Phenomenology is an appeal to experience if it is anything at all. To be clear, one should distinguish between an *a priori* claim (i.e., a claim that is not based on experience) and a claim about an *a priori* aspect of experience. Thus, when a phenomenologist claims that it is only in consultation with *one’s own* experience that one can identify an implicit SO, this implies, on the strongest interpretation, that such a thing is possible only because we, as human experiencers, have experiences that are *a priori* our own. Whether this strong claim is true or not, this is not an argument based on *a priori* assumptions; it’s based on an appeal to experience.

9 Di Francesco and colleagues rightly suggest that there are two forms of adultism (or what they call ‘adultocentrism’): the excluding kind (the infant is not like an adult), or the projecting kind (the infant is like an adult) (2016, p. 82). It’s not clear that they avoid the excluding form of adultism themselves since they make a lot of claims about precisely what infants are lacking in their experience (see, e.g., p. 85).

10 To be clear, in seeking empirical evidence for SO, as in many experiments and discussions the focus usually shifts to the sense of body ownership or SO for movement or action – this is *my* hand or this is *my* body, this is *my* movement – and this involves proprioception and kinaesthesia. It is specifically the implicit reflexive (or ecological, or auto- or self-awareness) character of proprioception (giving me information about or awareness of myself) that is at stake here, rather than the intentional or sensory content of the experience. As one reviewer notes, “the mineness-feature is constant across sensory experiences irrespective of the content.” In tactile experience, for example, I might touch different objects, X, Y, and Z, but the touching experience is always mine because of its proprioceptive nature. If X turns out to be my body (in the case of self-touch) proprioception is doubled (and, as Martin, 1995 suggests, body ownership is tied to body boundary, which is also proprioceptively defined). See Merleau-Ponty’s (2012) concept of ‘reversibility,’ discussed in the final section.

11 Gallese and Sinigaglia (2010), in their extensive of the empirical research, rightly associate this with the concept of motor intentionality. They claim that this sense of potentiality for action is more primitive and a necessary condition for both SO and SA. A more deflated account would suggest that as an aspect of bodily experience it is intrinsically integrated with SO and SA, part of what Merleau-Ponty (1964) would call a ‘form’ or gestalt structure of the minimal self.

12 We see this in the case of IW who, when he first was unable to control his bodily movement felt alienated from his body (Cole, 1995).
